# In Ovo Exposure to Phenanthrene Promotes Antimicrobial Resistance Genes and Hemolysin Virulence Factor in Guts of Chicken Embryos

**DOI:** 10.3390/toxics14100849

**Published:** 2026-09-24

**Authors:** Yaoxin Wang, Wenqian Yu, Kazuki Kanda, Hisato Iwata, Jiahua Guo

**Affiliations:** 1 Xi’an Key Laboratory of Environmental Simulation and Ecological Health in the Yellow River Basin, College of Urban and Environmental Sciences, Northwest University, Xi’an 710127, China; 202433439@stumail.nwu.edu.cn (Y.W.); 202421712@stumail.nwu.edu.cn (W.Y.); 2 Shaanxi Key Laboratory of Earth Surface System and Environmental Carrying Capacity, College of Urban and Environmental Sciences, Northwest University, Xi’an 710127, China; 3 Center for Marine Environmental Studies, Ehime University, Matsuyama 790-8577, Japan; kanda.kazuki.hi@ehime-u.ac.jp (K.K.); iwata.hisato.mz@ehime-u.ac.jp (H.I.)

**Keywords:** antimicrobial resistance gene, phenanthrene, chicken embryo, metagenomics, gut microbiome

## Abstract

Antimicrobial resistance genes (ARGs) are increasingly recognized as emerging environmental contaminants that threaten ecological integrity and animal health. Polycyclic aromatic hydrocarbons (PAHs) are widespread pollutants that accumulate in the environment and have been detected in wild bird eggs. Although PAHs have been shown to accelerate ARG dissemination in environmental matrices, their influence on ARG dynamics in birds is unclear. Therefore, it is hypothesized that exposure to elevated levels of PAHs promotes the propagation and enrichment of ARGs in the avian gut. In this study, chicken embryos at Hamburger–Hamilton Stage 1 (HHS 1) were exposed in ovo to phenanthrene (PHE; 1, 100, and 400 nmol/g egg), and the toxic effects were evaluated. High PHE exposure significantly reduced embryonic tarsus length by 14.81% and increased the liver somatic index (LSI) by 26.97%. Functional annotation based on the Virulence Factor Database (VFDB) showed that the relative abundance of hemolysin, a microbial virulence factor associated with gastrointestinal microorganisms, was significantly elevated in the high-PHE group. In a Kyoto Encyclopedia of Genes and Genomes (KEGG) analysis, genes involved in nucleotide excision repair (NER) and basal transcription factor (BTF) pathways showed significantly lower relative abundance following high-dose exposure. Additionally, ARGs related to polymyxin and pleuromutilin resistance were significantly enriched in the high-exposure group. Partial least squares path modeling (PLS-PM) indicated that ARG enrichment was jointly shaped by multiple factors. PHE may be directly associated with ARG proliferation or may act indirectly through mobile genetic elements (MGEs), whereas bacterial community abundance might be linked to the potential dissemination of ARGs given its significant positive association with MGEs. Collectively, these findings provide correlative evidence that PHE exposure is associated with increased ARG abundance in avian organisms and highlight the need for an integrated evaluation of PAH-associated hazards to avian health.

## 1. Introduction

Antimicrobial resistance has become a major global public health challenge and has intensified markedly since the beginning of the 21st century [1]. According to data reported in 2022, 23.78% of antimicrobial resistance genes (ARGs) were identified as posing biological health risks [2]. Owing to their ecological traits (e.g., feeding habits, migratory behavior, and trophic position), birds are regarded as key vectors for the dissemination of ARGs across environments and species [3]. For instance, the relative abundances of dominant ARGs and mobile genetic elements (MGEs) in the feces of common urban birds, including ducks, crows, and seagulls, reached 10^−5^–10^−1^ copies/16S rRNA and 10^−3^–10^−1^ copies/16S rRNA, respectively, which were comparable to those detected in feces from antimicrobial-fed poultry [4]. As the most important microbial niche in birds, the gut constitutes the principal site for ARG multiplication and dissemination [5], with horizontal gene transfer (HGT) being recognized as a central mechanism underlying ARG spread in the avian intestine [6]. In the guts of wild birds, plasmids constitute key members of MGEs, and diverse MGEs (e.g., the transposase gene (tnpA), insertion sequences (ISs), and integrons (intI1)) collectively serve as the major vectors for ARG transfer, with studies demonstrating that the risk of MGE-mediated HGT increases with trophic level [7]. In addition to antimicrobials, other micropollutants, such as microplastics, have been reported to drive ARG enrichment and transmission in the avian gut [8,9]. However, the effects of polycyclic aromatic hydrocarbons (PAHs) on the bird gut resistome remain underexplored.

Because of their persistence, bioaccumulation potential, and toxicity, PAHs are widespread organic pollutants in soil, air, and marine environments [10]. These compounds can induce inflammatory responses in the ileal and colonic mucosa by altering the structure of mouse gut mucosa-associated microbiota and impairing intestinal barrier integrity [11]. Consistently, studies on the human gut microbiota have shown that PAHs can dysregulate the transcription of multiple metabolic pathways and disrupt normal microbial function [12]. Although PAHs tend to accumulate in the avian gut rather than in other tissues [13], their gut toxicity in birds has received limited attention. More importantly, PAHs have been implicated in accelerating ARG dissemination within microbial communities. In an indoor microcosm experiment, PAH exposure markedly increased the abundances of sulfonamide- and aminoglycoside-resistance genes, as well as other ARGs in coastal sediments, an effect attributed to MGE-mediated HGT [14]. Similarly, the ARG abundance at petrochemical-contaminated sites with high PAH levels was reported to be 15 times that in pristine soil [15]. Comparatively high PAH burdens have also been recorded in avian species from several Asian countries. For example, gonadal tissues of *Mergus squamatus* collected from Poyang Lake, China, contained a maximum total PAH concentration of 1609 ng/g dry weight, with PHE as the predominant congener [16]. In Ahmedabad, Gujarat, India, a maximum concentration of 2037 ng/g wet weight was detected in the livers of endangered *Gyps bengalensis* [17]. In addition, PAHs can be deposited or “excreted” into eggs by female birds during yolk formation, and avian embryos are generally more sensitive than adults to environmental pollutants, exhibiting elevated susceptibility to mortality, malformation, and developmental retardation under PAH exposure [18,19]. The initial gut microbiota of avian embryos is vertically acquired from maternal oviduct microbes via egg white transmission [20]. Therefore, it is necessary to evaluate gut-associated risks and ARG transmission during avian embryonic development following PAH exposure.

Conventional methods for ARG detection have relied mainly on quantitative real-time PCR (qPCR). However, this approach is constrained by several limitations, including long assay duration, strict dependence on specific primers, and inability to detect unknown ARGs [21]. Compared with traditional qPCR-based assays, metagenomic next-generation sequencing (mNGS) has emerged as a powerful strategy for the comprehensive characterization of the avian gut microbiome and resistome while also enabling the identification of associations between ARGs and MGEs [22,23]. This approach has been used to characterize the gut microbiome and resistome profiles in migratory birds and across different stages of laying hens [5,24]. In contrast, corresponding evidence from chicken embryos, which are widely recognized as model biosystems for investigating avian embryonic development, remains unavailable.

In this study, an in ovo exposure assay was conducted to assess the effects of prenatal exposure to phenanthrene (PHE), a representative PAH pollutant, on the gut microbiota and resistome of chicken embryos. Our study assumed that PHE exposure could promote MGE-mediated horizontal transfer of ARGs in the embryonic gut by altering gut microbial abundance and diversity. The objectives of this study were threefold: (1) to determine how PHE exposure affects the abundance and functional profiles of the gut microbial community in chicken embryos, with particular emphasis on virulence-related and metabolic functions; (2) to characterize the gut resistome of chicken embryos; and (3) to clarify the intrinsic relationships among the microbiome, ARGs, and MGEs under PHE exposure.

## 2. Materials and Methods

### 2.1. Chemical Solution Preparation, In Ovo Injection, and Embryonic Tissue Measurement

Phenanthrene (PHE; ≥99.5%, 695114-1G) was purchased from Merck (Tokyo, Japan). DMSO (99.8%; CAS no. 67-68-5) was obtained from Wako Pure Chemical Industries (Osaka, Japan). For the dosing preparation, PHE was first dissolved in a small amount of DMSO, and the resulting stock solution was serially diluted with DMSO until the target concentration was reached.

Rhode Island Red chicken eggs (*Gallus gallus domesticus*) at HHS 1 (day 0 of incubation) were supplied by Yamagishi Jikkenchi (Tsu, Japan). All eggs were obtained from a single breeder flock, where hens were raised under identical feed and husbandry conditions to minimize differences in the maternal microbiota among individual eggs. Following egg weighing and candling, a small opening (approximately 1 mm in diameter) was created at the center of each shell using a metal probe. The chemicals were injected using a fine pipette, after which the opening was sealed with a small amount of molten sterile paraffin [25]. Based on the reported mean PHE concentration (342.16 ± 217.90 μg/kg dry weight) in environmentally disturbed wild avian eggs [26], 1 nmol/g egg PHE was set as an environmentally relevant exposure scenario, and doses of 100 nmol/g egg and 400 nmol/g egg were used to investigate dose-dependent effects on gut microbiota, ARGs, and microbial functions. Before injection, fertilized eggs were randomly assigned to four exposure groups using the sample function in R (v4.5.1). The grouping scheme was as follows: control (0.1% DMSO, n = 30), low PHE (1 nmol/g egg, n = 30), medium PHE (100 nmol/g egg, n = 30), and high PHE (400 nmol/g egg, n = 20). The injection volume for each egg was calculated by scaling a reference volume of 10 μL based on the ratio of individual egg weight to the mean egg weight of 60 g (injection volumes: 9.75–11.01 μL) to administer DMSO and the target PHE doses. Fewer eggs were allocated to the high-PHE group on the premise that sufficient valid samples for subsequent analyses could be secured, because high concentrations of PHE may lead to embryonic mortality and potential cross-contamination between samples. After injection, all embryos were incubated with automatic rotation (1 cycle per hour) in a chamber maintained at 55 ± 2% relative humidity and 38 ± 1 °C before hatching (Hamburger–Hamilton Stage, HHS 44-45).

At HHS 44-45, non-viable and severely malformed embryos were excluded first. Eight embryos per group were then randomly selected from the control and each exposure group using the sample function in R (v4.5.1). These selected embryos were subjected to phenotypic assessment. The measured biological and morphological variables included body weight (yolk sac-free weight), tarsus length (measured from the posterior part of the intertarsal joint to the distal end of the tarsus), upper bill length, lower bill length, and liver weight [27]. In addition, the bill index (upper bill length/lower bill length × 100%) and liver somatic index (LSI = liver weight/embryo weight × 100%) were calculated for each embryo and used to evaluate health condition and morphological response [28,29].

### 2.2. DNA Extraction, Metagenomic Sequencing, and Basic Bioinformatics Analysis

Cecal samples collected from the 32 selected chicken embryos (eight embryos per group, as described above) were used for shotgun metagenomic sequencing and subsequent bioinformatic analysis. Under aseptic conditions, both ends of the cecum were cut open with a sterile scalpel. The cecal contents were collected by rinsing the intestinal segment with sterile water. The recovered contents were transferred into 2 mL sterile centrifuge tubes and centrifuged at 10,000 *g* for 5 min at 4 °C to harvest the precipitate. The precipitates were aliquoted into sterile centrifuge tubes, frozen in liquid nitrogen for more than 10 min, and subsequently stored at −80 °C for subsequent DNA extraction. Microbial genomic DNA was isolated using a Nucleospin Tissue DNA Kit (Takara, Kusatsu, Japan), and the DNA concentration and purity were determined using a NanoPhotometer N60 (Implen, Munich, Germany). The extracted DNA samples were submitted to Novogene Co., Ltd. (Tokyo, Japan) for sequencing. The library was constructed using the ALFA-SEQ DNA Library Prep Kit (Novogene, Tokyo, Japan), incorporating index labels. After library quality assessment using a Qubit 4.0 Fluorometer (Thermo Fisher Scientific, Waltham, MA, USA) and QSEP400 system (Houze Bio-Tech, Hangzhou, China), sequencing was performed on an Illumina NovaSeq 6000 platform (Novogene, Tokyo, Japan) to generate 150 bp paired-end reads [30]. For raw data processing, low-quality sequences were trimmed and removed using Trimmomatic (v. 0.36), yielding clean reads for downstream analyses. Metagenome assembly for each sample was conducted using MEGAHIT (v. 1.0.6), and N-free scaftigs ≥ 500 bp were retained after scaffold fragmentation. Open reading frames (ORFs) were subsequently predicted using MetaGeneMark (v. 3.38). Following redundancy removal with CD-HIT (v. 4.7), ORFs meeting the thresholds of 95% sequence similarity and 90% coverage were retained for further analyses [31]. Taxonomic annotation was performed by aligning sequences against the NCBI NR database using DIAMOND (v2.1.11), and hits with E-values ≤ 1 × 10^−10^ were preserved for taxonomic classification. Species-level annotation was assigned using the LCA algorithm implemented in MEGAN software (version 1.2.9) [32].

### 2.3. Annotation and Quantification of ARGs and MGEs

The sequences corresponding to ARGs and MGEs in the metagenomic datasets were annotated and classified using the ARGs-OAP Online Analysis Pipeline (version 3.2.4). For preliminary identification, the segmented sequences were aligned against the structured ARG (SARG) database for ARGs and the corresponding MGE reference databases with USEARCH-ublast under the criteria of e-value < 1 × 10^−10^ and sensitivity > 0.5, screening the sequences associated with ARGs and MGEs. In parallel, sequencing reads were mapped to the Greengene database to retrieve 16S rRNA gene sequences and estimate their copy numbers [33]. To determine the cell number, split fa sequences were aligned against the in-house KEGG screening database using DIAMOND with thresholds of e-value ≤ 3 and identity ≥ 0.45. The sequence assignment was verified using BLASTX (Version 2.16.0) (e-value < 1 × 10^−7^, identity > 0.8), after which the validated sequences were classified into individual subtypes. Finally, the gene abundances derived from the above procedures were normalized, and tags per million (TPM) was adopted as the unit for quantitative standardization.

### 2.4. Co-Occurrence Network Analysis

To construct the co-occurrence network, the top 50 ARG subtypes, MGE subtypes, and gut microbial taxa ranked by abundance were selected under the thresholds of *p* < 0.01 and |R| > 0.8, and the resulting network was visualized using Cytoscape (v. 3.9.1). In addition, a species-species interaction network was generated for the top 100 gut microbes based on abundance. Subsequently, the nodes showing both betweenness centrality and degree values higher than the corresponding network averages were screened and defined as hub species.

### 2.5. Statistical Analysis

Differences in morphological development indices, microbial taxonomic abundance, and functional categories annotated from the Virulence Factor Database (VFDB) and Kyoto Encyclopedia of Genes and Genomes (KEGG) were evaluated statistically. The Shapiro–Wilk test was used to assess the normality of data distributions, and Levene’s test was applied to evaluate homogeneity of variance. For datasets satisfying both normality and homogeneity of variance assumptions, one-way analysis of variance (ANOVA) followed by Dunnett’s post hoc test was adopted. For datasets with a normal distribution but unequal variances, Welch’s ANOVA followed by the Games–Howell post hoc test was performed. When the normality assumption was violated, the nonparametric Kruskal–Wallis test followed by Dunn’s post hoc test was performed. Heatmaps generated using the heatmap package were used to display ARG and MGE abundance patterns across the four PHE exposure groups. Venn diagrams of ARGs were constructed using Origin (v. 2024). To characterize shifts in microbial community diversity under PHE exposure, species diversity indices were calculated using the Magigene Cloud Platform (Magigene Biotechnology, Guangzhou, China). Partial least squares path modeling (PLS-PM) was performed in R software (v. 4.5.1) using the plspm and vegan packages.

## 3. Results

### 3.1. Evaluation of Embryonic Phenotypes

In the in ovo exposure experiment, embryo survival varied among the treatment groups subjected to different PHE concentrations. The survival rates were 86.7% (26/30), 83.3% (25/30), 93.3% (28/30), and 75.0% (15/20) in the control, low-PHE, medium-PHE, and high-PHE groups, respectively. Phenotypic assessment demonstrated that tarsus length was significantly reduced in embryos from the high-PHE group (Figure 1b), corresponding to a 14.81% decrease compared with that in the control group. In addition, LSI was significantly increased in both the medium- and high-PHE groups (Figure 1d), with an increase of 26.97% in the high-dose group. Although the mean body weight of embryos in the high-PHE group (23.99 ± 4.07 g) was lower than that in the control group (28.03 ± 1.67 g), no statistically significant difference was observed. No significant variations in body weight or bill index were detected among all groups (Figure 1a,c).

### 3.2. Community Characteristics and Functional Differences in Gut Microbiota

Significant shifts in gut microbial composition were observed between the control and PHE-treated groups (Figure 2a). At the species level, *Sphingomicrobium* sp. was significantly enriched only in the low-PHE group, whereas this increase was not maintained under medium or high exposure. In contrast, *Novosphingobium nitrogenifigens* showed a significant reduction in the high-PHE group (Figure 2b). In addition, the relative abundances of *Chlamydia psittaci* and *Chlamydia abortus* tended to increase under high PHE exposure, although the differences were not statistically significant. Co-occurrence network analysis demonstrated that nearly 98% of the species in the chicken embryo gut microbiota were positively correlated, suggesting a tightly associated microbial community structure (Figure S1). Cluster analysis also revealed evident differences in hub taxon composition among the treatment groups. In the control group, *Pseudomonadota bacterium* was identified as the dominant species. Compared with the control, unclassified *Microbacteriaceae* emerged as a dominant taxon unique to both the low- and high-PHE groups, *Pseudomonas aeruginosa* was specifically dominant in the medium- and high-PHE groups, and *Escherichia coli* was detected as a dominant species only in the medium-PHE group.

Substantial functional differences were detected among the PHE treatment groups at the levels of VFDB virulence factors and KEGG pathways (Figure 3). In the VFDB-based analysis (Figure 3a), the relative abundance of hemolysin (encoded by the gene *hlyA*) increased progressively with increasing PHE concentration and was significantly elevated in the high-PHE group. In contrast, the relative abundance of *ompA* was markedly reduced in the medium-PHE group. KEGG pathway analysis (Figure 3b) indicated a gradual decline in the relative abundances of the nucleotide excision repair (NER) and basal transcription factor (BTF) pathways with increasing PHE concentration, with a significant reduction observed under high-dose exposure. Metagenomic analysis observed eight orthologs associated with the NER pathway (*uvrA*, *uvrB*, *uvrC*, *uvrD*, *polA*, *ligA/ligB*, *mfd*, *ERCC2/XPD*). *ERCC2/XPD* was the only ortholog detected for the BTF pathway, which encodes a shared TFIIH-associated helicase subunit.

### 3.3. Co-Occurrence Characteristics of ARGs with MGEs and the Microbial Community, as Well as the Key Drivers of ARGs

A total of 23 ARG types and 179 ARG subtypes were recovered from the metagenomic datasets of chicken embryo gut samples. The composition was dominated by multidrug resistance genes (*oprM* was the most abundant subtype, followed by *muxB*) and bacitracin resistance genes (*bacA* was the most abundant subtype, followed by *bcrA*), which accounted for 41.07% and 7.30% of the total ARG abundance, respectively. The subsequent major categories were quinolone resistance genes (*abaQ* was the most abundant subtype, followed by *lfrA*), with 6.41% of the total ARG abundance; aminoglycoside resistance genes (*aph(6)-Id* was the most abundant subtype, followed by *aph(3″)-Ib*), with 6.17%; and polymyxin resistance genes (*rosA* was the most abundant subtype, followed by *arnA*), with 5.81%. Of these ARGs, 52 subtypes were shared among all four treatment groups (Figure S2), indicating that a core set of intrinsic resistance determinants was stably present in the gut microbiota. Group-specific ARG subtypes were detected in every treatment, and the largest number occurred in the high-PHE group, in which 35 unique subtypes were identified, compared with 13, 19, and 12 in the control, low-, and medium-PHE groups, respectively. The proportion of unique ARGs was the highest in the high-dose group, reaching 38.6%. In addition, pronounced individual variation in ARG subtype abundance was evident among samples (Figure 4a). Among the significantly altered ARGs (Figure 4b,c), *rosA*, a polymyxin resistance gene, and *TaeA*, a pleuromutilin–tiamulin resistance gene, were both significantly enriched under high PHE exposure compared with the control. In contrast, the multidrug resistance gene *smeF* was significantly upregulated only in the low-dose group, whereas *smeE* consistently declined across all PHE-treated groups.

Only two MGE types and ten MGE subtypes were detected in this study (Figure 4a), with transposases representing the dominant category and IS elements occurring at high abundance. Among these MGEs, *tnpA* was the most abundant transposase subtype, whereas *IS91* was the dominant insertion sequence subtype. Notably, several highly abundant MGE subtypes displayed distribution patterns similar to those of ARG subtypes enriched in the same samples, suggesting a potential co-occurrence between the two groups. Although the difference was not statistically significant, the MGE abundance in the high-PHE group exceeded that in the control group (Figure 4c).

Network correlation analysis demonstrated that ARGs, MGEs, and the gut bacterial community were tightly interconnected under PHE exposure (Figure 5). The control, low-, medium-, and high-PHE groups contained 39, 59, 40, and 58 nodes, respectively, which were connected by 31, 77, 27, and 75 edges. Compared with the exposed groups, the control network was less densely connected, indicating weaker associations among ARGs, MGEs, and bacterial taxa in the absence of PHE treatment. Moreover, the transposase along with the insertion sequences *IS91* and *istA* remained relatively abundant in multiple samples and demonstrated positive associations with ARGs such as *APH(3)-Ib*, *TaeA*, and *ileS*. A marked expansion of potential ARG hosts was also observed in the chicken embryo gut after high-dose PHE exposure. Compared with the control group, the high-PHE group contained a substantially higher number of host species (Figure 5a,d), with 35 host taxa distributed across three phyla. Among these, *Proteobacteria* and *Actinobacteriota* were the principal ARG reservoirs. *Herbaspirillum* co-occurred with numerous ARGs in the control, low-, and high-PHE groups, whereas *Sphingomonas* was repeatedly detected in the low- and high-PHE groups. The shared host taxa were present across all treatments, most notably *Pseudomonas* within the phylum *Proteobacteria*, which was consistently detected in every group.

The PLS-PM results showed that ARG enrichment was not driven by a single factor but emerged from direct and indirect regulatory pathways (Figure S3). PHE exerted a significant direct promoting effect on ARG abundance, and an additional indirect effect was mediated through PHE-induced shifts in the microbial community structure. Species abundance significantly increased MGE abundance, which enhanced ARG levels. In contrast, microbial community diversity was negatively associated with ARGs, compared with the positive contribution of MGEs.

## 4. Discussion

In the present study, in ovo PHE exposure was found to induce developmental toxicity in chicken embryos. Metagenomic profiling suggested that microbial community remodeling, virulence-related changes, and ARG enrichment did not follow a strict dose-dependent pattern. The results suggest that the persistence and dissemination of ARGs in the embryonic gut were jointly shaped by external pollutant pressure and gut microbial dysbiosis, pointing to the possibility of interbacterial gene transfer via MGE-mediated horizontal gene transfer. These findings partially align with our initial hypothesis that high PHE exposure could disrupt the structure of the embryonic gut microbiota, which would favor MGE-associated ARG enrichment and raise the risk of ARG dissemination within the avian gut. The following sections focus on microbial community structure and function, ARG host composition, and the mechanistic basis of ARG propagation under PHE contamination.

### 4.1. PHE Exposure Reshapes the Gut Microbial Community Structure in Chicken Embryos

The capacity of the gut microbiota to tolerate PHE appears to be supported by several dominant microbial phyla. In total, 11 phyla were detected, among which *Pseudomonadota* (72.3%), *Chlamydiota* (17.6%), *Basidiomycota* (5.3%), and *Actinomycetota* (3.8%) were the dominant groups. Among them, *Pseudomonadota* (formerly known as *Proteobacteria*) appeared to function as the central phylum associated with PHE degradation. Its affiliated genus *Pseudomonas* can degrade PHE through the lateral dioxygenase pathway while simultaneously secreting glycolipid and lipopeptide biosurfactants that enhance PHE solubility and bioavailability in the gut [34]. *Basidiomycota* and *Actinomycetota* can serve as auxiliary phyla by jointly decomposing organic substrates to obtain carbon sources. Specifically, *Actinomycetota* can transform PHE intermediates through secondary metabolism, whereas *Basidiomycota* can directly degrade PHE [35,36]. In contrast, *Chlamydiota* comprises obligate intracellular microorganisms that depend on eukaryotic host cells for nutrients and energy [37]. One representative species, *Chlamydia psittaci*, can colonize the chicken gut, replicate efficiently in gut mucosal epithelial cells and macrophages, spread through cloacal excretion, and interact with gut microorganisms, such as pathogenic *Escherichia coli* [38]. Meanwhile, PHE stress may aggravate gut mucosal injury and impair barrier integrity [39], thereby indirectly altering the colonization and metabolic efficiency of PHE-degrading microbiota and disturbing gut microecological homeostasis.

The responses of specific bacterial taxa further support this interpretation. As a representative aromatic compound-degrading bacterium [40], *Sphingomicrobium* sp. can utilize PHE as a carbon and energy source [41,42], which may explain its competitive advantage in the microbial community. However, its abundance was suppressed under high PHE exposure, suggesting that excessive PHE toxicity may exceed its degradative capacity and disrupt cellular homeostasis. A comparable pattern was observed during the degradation of 3-phenoxybenzoic acid by the typical *Sphingobium* strain *Sphingobium baderi* SC-1 [43]. *Novosphingobium nitrogenifigens* is a typical nitrogen-fixing microorganism [44]. Its sensitivity to PHE may reflect an energetic trade-off between nitrogen fixation and PAH detoxification because the high energy demand of nitrogen fixation can make this bacterium more vulnerable to PHE-induced cytotoxicity. This interpretation is consistent with the observations of studies examining the effects of aromatic compounds on *Rhodopseudomonas* [45]. Under high-concentration stress, PHE-tolerant degraders may become selectively dominant, whereas PHE-sensitive taxa decline in abundance.

### 4.2. Genetic Alterations in Microbial Functional Groups Under PHE Contamination

Hemolysin, which is widely distributed among avian gut microorganisms [46], may contribute indirectly to ARG transmission by disrupting host cellular barriers [47]. Furthermore, hemolysin is involved in the transition of bacteria from environmental organisms to host-adapted pathogens, as illustrated by *Vibrio cholerae* [48]. For example, hemolysin produced by murine uropathogenic *Escherichia coli* (UPEC) enhances pathogenicity by disrupting host cell membrane integrity and inducing necrosis, thereby facilitating bacterial adaptation to the host microenvironment [49]. In this context, the elevated abundance of hemolysin observed in the present study may reflect an adaptive microbial response to PHE stress. It should be noted that our metagenomic data only reflect the increased relative abundance of hemolysin virulence factor sequences and do not confirm elevated production of biologically active hemolysin protein. In contrast, the reduced abundance of *ompA* in the medium-PHE group suggests that gut microorganisms may suppress the expression of this virulence-associated gene under PHE exposure to conserve energy and redirect limited resources towards PHE degradation and detoxification. This interpretation is supported by previous studies showing that microorganisms can reallocate energy under environmental stress and downregulate virulence-associated genes to sustain essential survival processes, including carbon source utilization and intracellular magnesium homeostasis [50,51]. In addition, the downregulation of *ompA* may weaken the adhesive and invasive capacities of Gram-negative bacteria, thereby reducing damage to the intestinal mucosal barrier, representing a defensive adaptive response. Consistent with this interpretation, knockout of *ompA* in *Salmonella* Typhimurium has been shown to markedly attenuate adhesion to and invasion of murine gut epithelial cells [52].

The NER pathway is a major repair system for DNA damage induced by exogenous pollutants such as PAHs [53]. In the present study, the significant decline in the abundance of NER-related genes suggests that DNA damage in the gut could be aggravated as PHE exposure increases. A similar pattern has been reported in the human reproductive system, where elevated PAH exposure, assessed using urinary 1-hydroxypyrene as a biomarker, is associated with increased sperm DNA damage linked to the NER pathway [54]. The observed downregulation of NER-associated genes may be attributable to PHE-induced disruption of the structural integrity of key genes, suppression of gene expression regulation, and impairment of repair enzyme activity. This compromises the maintenance of normal NER function. Notably, exogenous PAHs have been shown to interfere with the NER pathway through these mechanisms [55]. The BTF pathway supports basal cellular transcription by assembling the preinitiation complex (PIC) (e.g., TFIIA, TFIIB, and TBP) and contributes to the maintenance of steady-state transcription of genes associated with nutrient absorption in gut cells [56,57]. From this perspective, PHE, as a PAH-type AhR ligand, may activate AhR and promote its heterodimerization, which can interfere with PIC function and reduce the abundance of the BTF pathway in the chicken gut. This interpretation is consistent with previous evidence showing that ligand-activated AhR forms a heterodimer with ARNT and represses basal transcription through direct interaction with BTF components, including TFIIF and TBP [58].

### 4.3. Hosts of ARGs in the Chicken Embryo Gut Under PHE Stress

Pathogenic bacteria carrying ARGs in the avian gut may represent an important threat to public health [59]. Under PHE exposure, pathogenic taxa such as *Chlamydia psittaci*, *Chlamydia abortus*, and pathogenic *Escherichia coli* were widely detected in the gut. *Chlamydia psittaci* can cause psittacosis in humans, which can rapidly progress to acute respiratory distress syndrome (ARDS) and multilobe lung lesions in severe cases, while showing poor responsiveness to conventional β-lactam therapy [60]. *Chlamydia abortus* can infect immunocompetent individuals via avian excreta, with clinical manifestations of high fever and progressive dyspnea, and is only sensitive to doxycycline [61]. Pathogenic *Escherichia coli* carries multiple virulence genes and often exhibits multidrug resistance. It can spread through contaminated poultry meat or water sources and typically causes diarrhea and abdominal pain, with systemic infection developing in severe cases [62]. Overall, the transmission and enrichment of ARGs by these pathogenic bacteria under PAH contamination suggest that PHE may impose selective pressure that favors antimicrobial-resistant pathogens, thereby promoting the prevalence and spread of high-risk ARG-carrying bacteria in the gut microbiota [15].

Under high-concentration PHE exposure, the abundances of *rosA*, *taeA* and *smeF* increased significantly. Among them, *rosA* represents a polymyxin resistance subtype widely distributed in environmental resistomes. Similarly, metagenomic studies on Antarctic glacier foreland resistomes have also identified it as a predominant polymyxin resistance subtype [63]. *rosA* is a component of the *rosAB* efflux-potassium antiporter system, which mediates bacterial resistance to cationic antimicrobial peptides such as polymyxins [64]. Meanwhile, resistomic analyses of *Chryseobacterium* have revealed the frequent occurrence of *rosA*, which provides a potential explanation for the previously reported colistin-resistant phenotypes within this genus [65]. *taeA* is an ABC transporter composed of a single polypeptide chain. Heterologous expression experiments demonstrated that cloning *taeA* into *E. coli* confers resistance to pleuromutilin antimicrobials on the host [66]. *smeF* encodes the outer membrane channel component of the multidrug resistance efflux system *smeDEF*. Studies have shown that cloning the full-length *smeDEF* operon into drug-susceptible *E. coli* Δ*acrAB* confers a multidrug-resistant phenotype against tetracycline, macrolides, and quinolone antimicrobials on the host [67]. Under PHE stress conditions, the abundance of the *smeF* gene in the gut microbiota of chicken embryos is upregulated, which may contribute to the development of multidrug resistance. The abundances of polymyxin- and pleuromutilin-associated ARGs exhibited the strongest responsive changes upon PHE exposure. Polymyxins and pleuromutilins are widely used in poultry for prophylaxis and treatment of bacterial infections of the gut and respiratory tract [68,69]. Notably, polymyxins also serve as the last-line antimicrobials for severe drug-resistant bacterial infections in humans [70]. The elevated abundance of resistance genes against these antimicrobials under PHE exposure indicates that PHE stress may increase the transmission risk of ARGs in both poultry and human health contexts.

The predicted ARG hosts in the embryonic gut were mainly affiliated with *Herbaspirillum*, *Microbacterium*, *Pseudomonas*, and *Sphingomonas*. *Herbaspirillum* has been recognized as an important ARG host, and both environmental and clinical strains have been reported to carry resistance determinants [71]. Similarly, the genomes of the genus *Microbacterium* harbor multiple drug resistance genes and display a multidrug-resistant phenotype, consistent with reports of frequent ARG enrichment in immunocompromised individuals [72]. Evidence from river ecosystems has shown that both *Pseudomonas* and *Sphingomonas* can carry multidrug resistance genes and co-occur with diverse virulence factors, contributing to the formation of the microbial resistome and virulence reservoirs [73]. In addition, *Escherichia coli* was detected only in the medium- and high-PHE groups. As a core ARG host in the gut of livestock and poultry, this species can harbor multiple resistance genes and virulence factors, thus acting as a potential reservoir for gut-associated ARGs [74].

### 4.4. PHE Exposure Gradient as a Key Driver of ARGs

MGE-mediated HGT has been recognized as a plausible mechanism for ARG dissemination in gut systems, and our correlative results point to its potential contribution within the chicken embryo gut [75]. The PLS-PM results suggest that high bacterial diversity may suppress ARG spread by weakening the dominance of ARG-hosting taxa through interspecific competition [76,77]. In contrast, increased bacterial abundance may enlarge the pool of potential ARG hosts, elevate MGE abundance, and increase the frequency of contact between MGEs and ARGs, thereby increasing the potential risk for horizontal ARG transfer [78]. Moreover, PHE was significantly positively associated with ARG abundance, suggesting that it may act as an environmental stressor contributing to ARG enrichment in the chicken gut, though the underlying mechanism remains unclear.

## 5. Conclusions

This study systematically characterized the microbial functional groups, MGEs, and ARG profiles in the guts of chicken embryos exposed to different concentrations of PHE. Overall, PHE exposure was associated with a lower abundance of genes involved in the NER and BTF pathways in the KEGG database, indicating potential shifts in DNA-repair-related and basal-transcription-related gene pools. Notably, PHE exposure was associated with a higher abundance of the virulence factor hemolysin in the gut microbiota, suggesting a potential increase in bacterial pathogenic potential. In parallel, substantial enrichment was observed for ARGs associated with polymyxin and pleuromutilin resistance. These findings provide new insights for veterinary clinical medication strategies. Collectively, these findings indicate that PHE exposure is associated with increased ARG abundance and a higher abundance of hemolysin-encoding genes in the gut of chicken embryos, suggesting a potential risk for ARG dissemination and a potential shift toward higher bacterial pathogenic potential. The underlying mechanisms remain to be elucidated at the physiological, biochemical, and molecular levels. Nevertheless, these findings provide potential clues linking PHE exposure to ARGs and hemolysin and offer directions for future studies on the effects of PAHs on avian gut ARGs and hemolysin. In future work, investigation of the ARG proliferation mechanism will be performed via in vitro bacterial culture experiments and transcriptomic analysis. It should be acknowledged, however, that this study only adopted PHE as a representative PAH in single-contaminant exposure assays, whereas wild birds in natural habitats are commonly exposed to complex mixtures of co-occurring PAHs; whether other PAHs are also associated with the spread and enrichment of ARGs in avian hosts therefore warrants further investigation. In addition, despite uniform rearing conditions for the breeder flock, inherent individual differences in maternal microbiota could not be completely eliminated, which may confound the initial embryonic gut microbiota. While the present study focused on chicken embryos, the results provide biologically relevant evidence that may inform future assessments and control of avian health risks and environmental risk management.

## Figures and Tables

**Figure 1:**
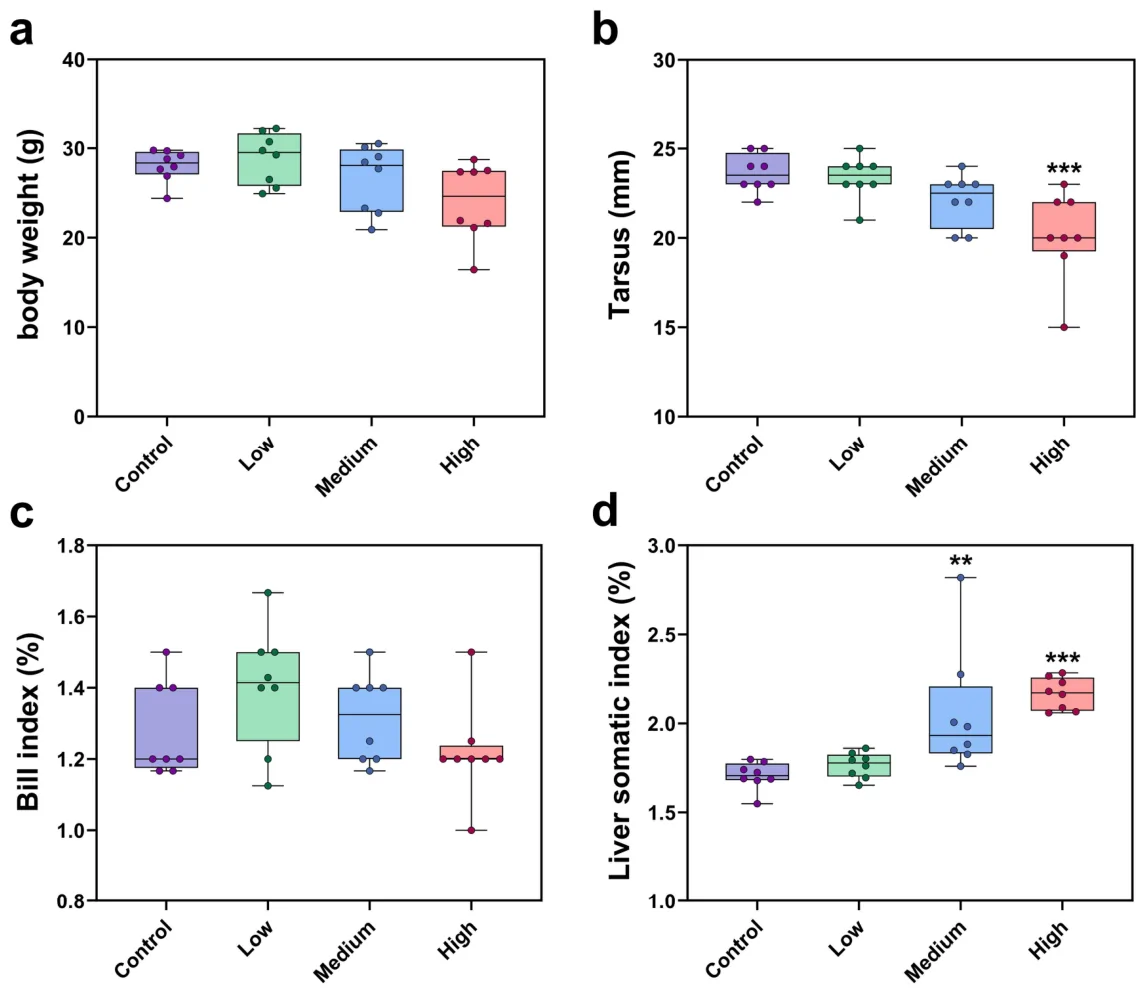
Effects of DMSO control and PHE exposure (low: 1, medium: 100, and high: 400 nmol g^−1^ egg) on growth and morphological indices of HHS 44-45 chicken embryos: (**a**) body weight, (**b**) tarsus length, (**c**) bill index, and (**d**) liver somatic index (LSI). (** *p* < 0.01, *** *p* < 0.001, n = 8 per group).

**Figure 2:**
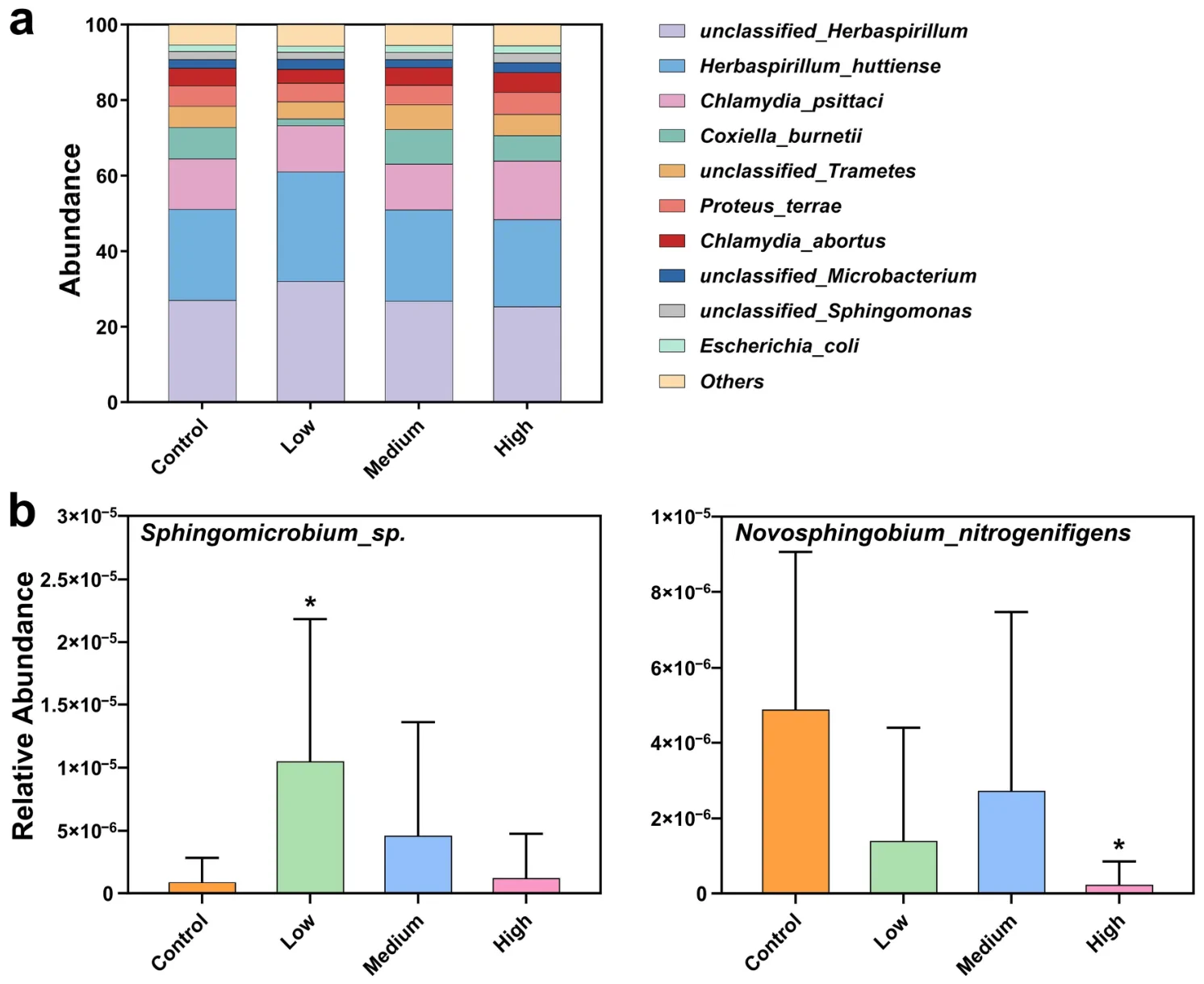
Species composition of chicken gut microbiota. (**a**) Relative abundance of the top 10 species across experimental groups. (**b**) Changes in the relative abundance of species with statistical significance (* *p* < 0.05, n = 8 per group).

**Figure 3:**
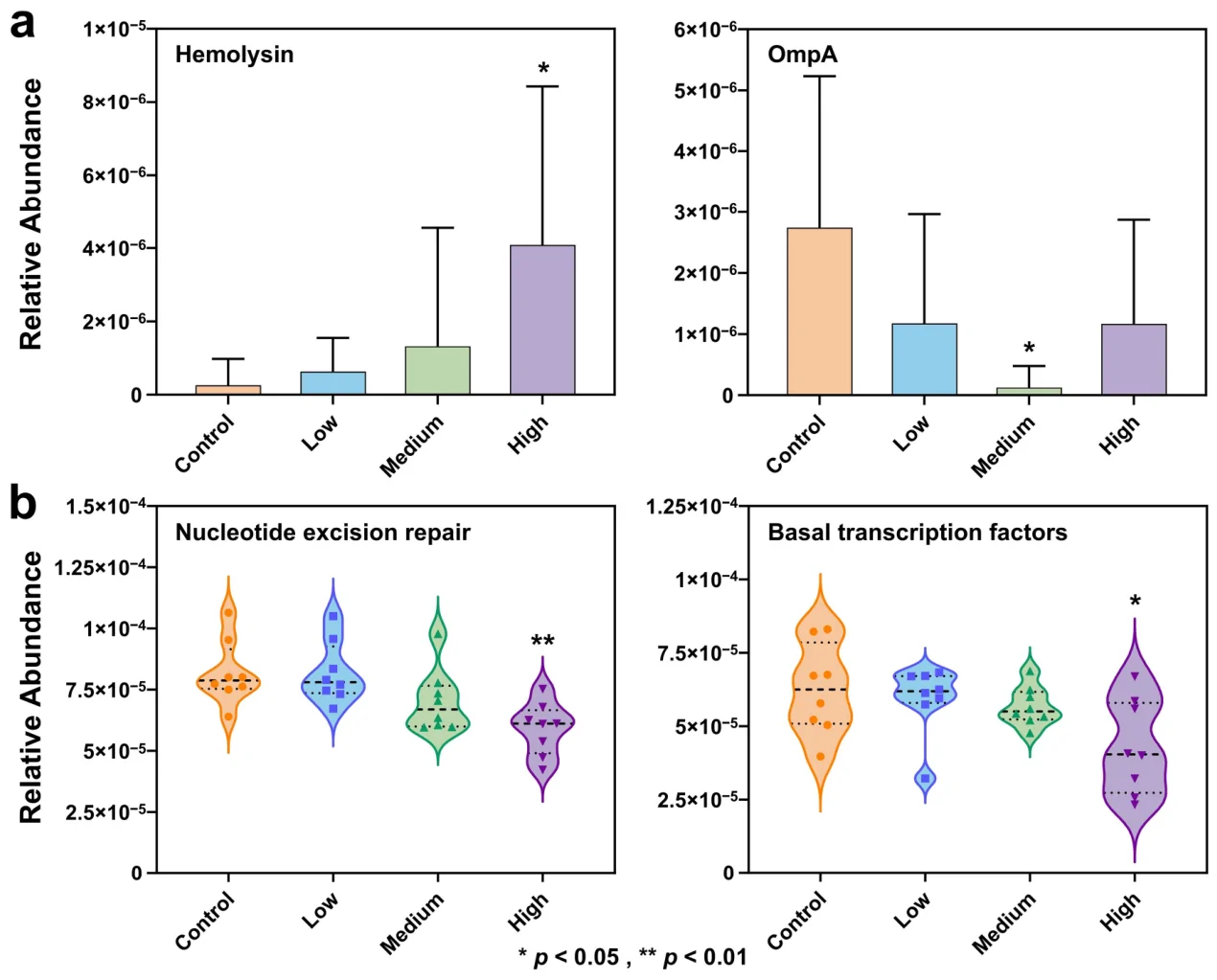
Functional annotation of the chicken gut microbial community. (**a**) Relative abundance of virulence factors based on VFDB annotation. (**b**) Relative abundance of functional pathways based on KEGG annotation. In panel (**b**), each violin plot shows the upper and lower dotted lines representing the 75th and 25th percentiles, respectively, and the dashed line inside denotes the median.

**Figure 4:**
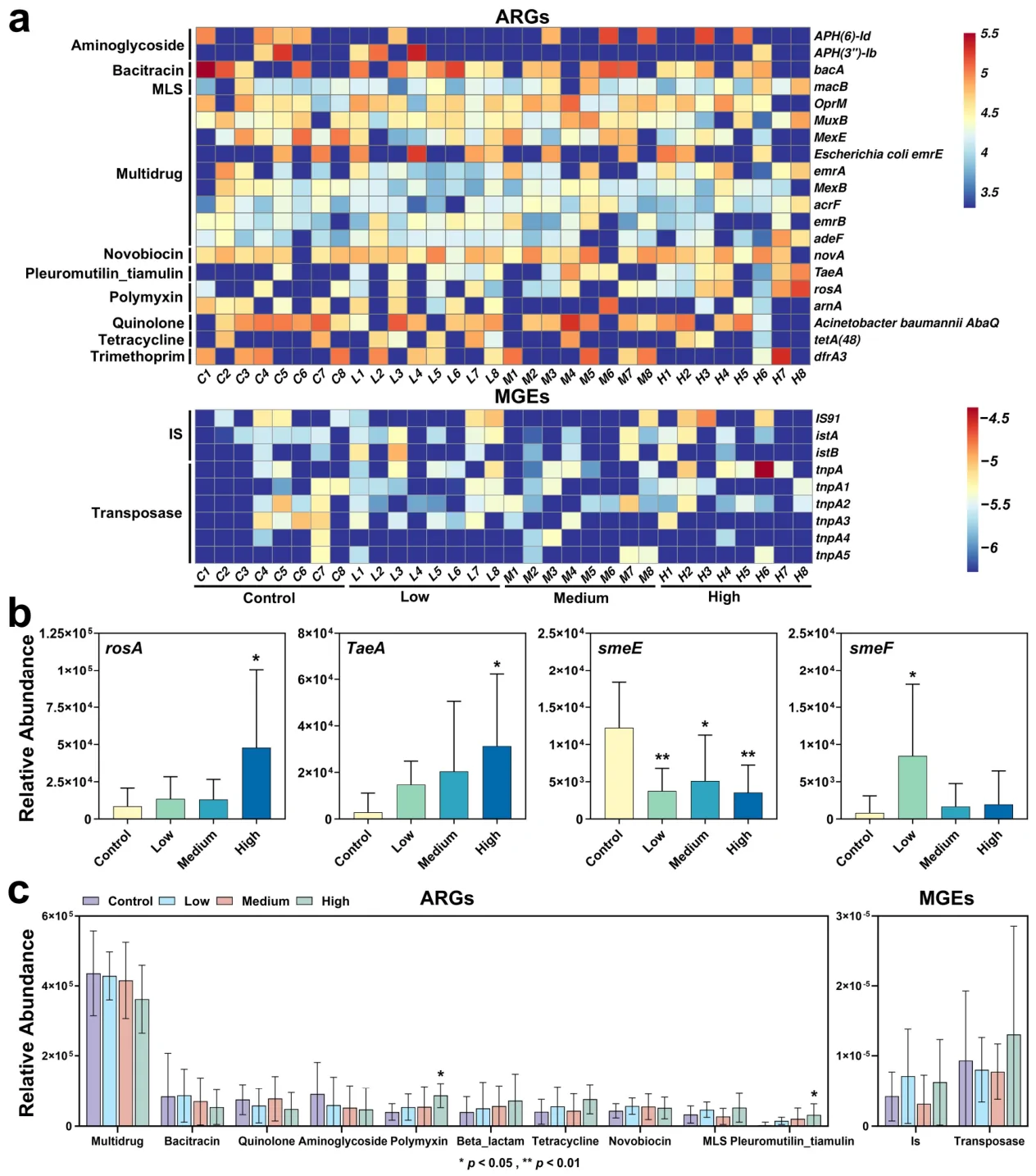
Abundance distribution of ARGs and MGEs in the control and PHE exposure groups. (**a**) Subtypes of ARGs and MGEs with their corresponding parent types. (**b**) Abundance of ARGs with significant differences. (**c**) Abundance profiles of the top 10 ARG types and all MGE types.

**Figure 5:**
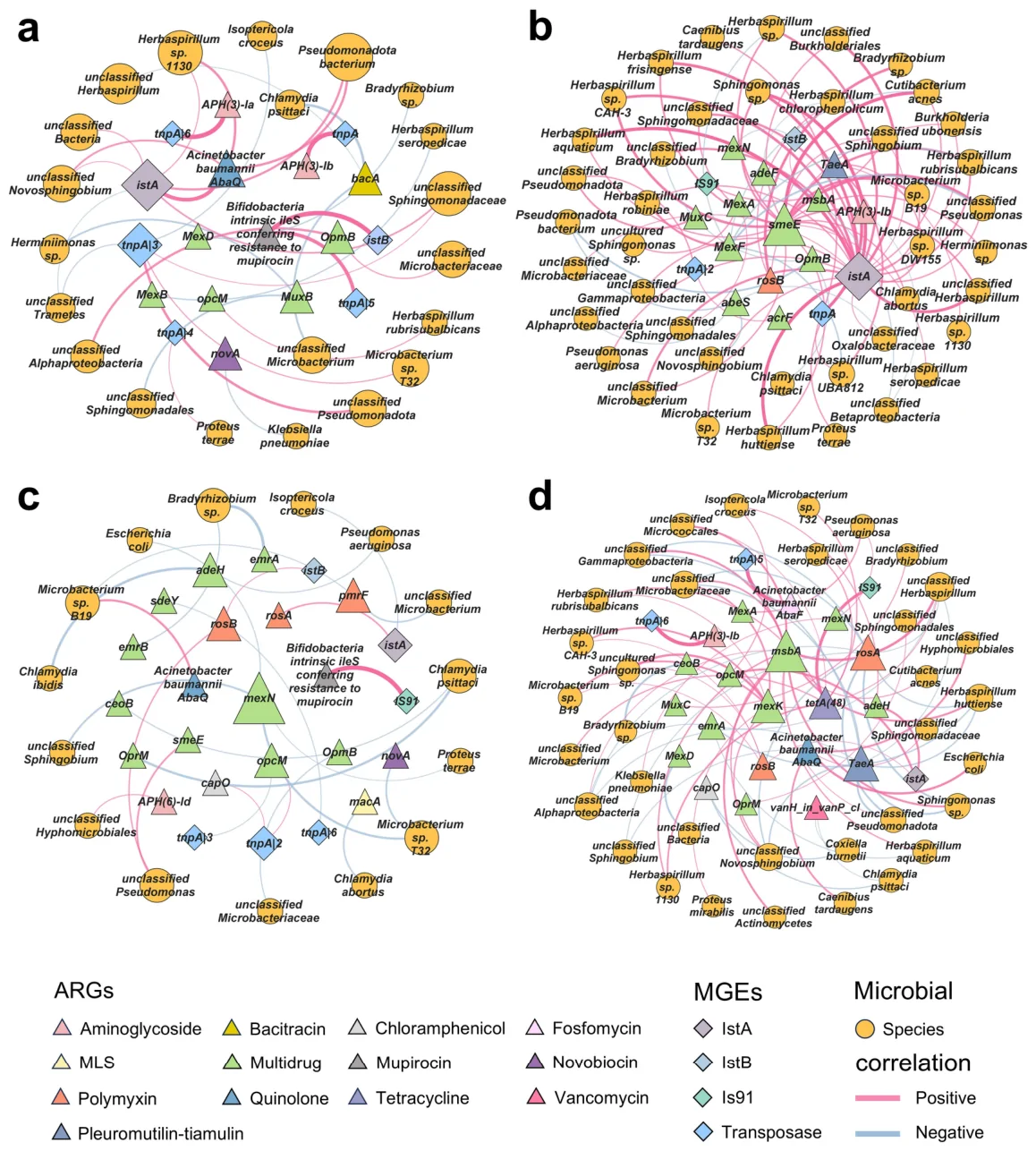
Co-occurrence network analysis illustrating associations among ARGs, MGEs, and the bacterial community in the (**a**) control, (**b**) low-, (**c**) medium-, and (**d**) high-PHE groups. Edge thickness indicates the correlation strength, and node size reflects the degree of connection. Only robust and statistically significant Spearman correlations are shown (|r| > 0.8, *p* < 0.01).

## Data Availability

The data presented in this study are available on request from the corresponding author.

## Supplementary Materials

The following supporting information can be downloaded at https://www.mdpi.com/article/10.3390/toxics14100849/s1, Figure S1: Co-occurrence patterns of the intestinal microbiota of chicken embryos in (**a**) control, (**b**) low, (**c**) medium and (**d**) high groups. The width of connecting lines indicates the magnitude of correlation, and the size of each node is proportional to the degree of connectivity. Only significant and robust Spearman correlations are displayed in the network (|R| > 0.8, *p* < 0.05); Figure S2: Venn diagram comparison of the number of ARGs; Figure S3: PLS-PM analysis of ARG abundance drivers. (**a**) Pathways linking PHE, bacterial community, bacterial diversity, MGEs, and ARGs. Positive and negative effects are indicated by red and blue, respectively. R^2^ values represent the explained variance. Abbreviations: B-abu, bacterial abundance; B-div, bacterial diversity. (* *p* < 0.05, ** *p* < 0.01, *** *p* < 0.001). (**b**) Effect sizes of the PLS-PM model.

## Abbreviations

The following abbreviations are used in this manuscript in alphabetical order:

APEC	Avian pathogenic *Escherichia coli*
ARDS	Acute respiratory distress syndrome
ARGs	Antimicrobial resistance genes
BTF	Basal transcription factor
HGT	Horizontal gene transfer
HHS	Hamburger–Hamilton stage
IntI1	Integrons
ISs	Insertion sequences
KEGG	Kyoto Encyclopedia of Genes and Genomes
MGEs	Mobile genetic elements
mNGS	Metagenomic next-generation sequencing
NER	Nucleotide excision repair
ORFs	Open reading frames
PAHs	Polycyclic aromatic hydrocarbons
PHE	Phenanthrene
PIC	Preinitiation complex
PLS-PM	Partial least squares path modeling
qPCR	Quantitative real-time polymerase chain reaction
R	Spearman’s rank correlation coefficient
SARG	Structured antimicrobial resistance gene database
tnpA	Transposase gene
TPM	Tags per million
UPEC	Uropathogenic *Escherichia coli*
VFDB	Virulence Factor Database
