# Supplementary material for: In Ovo Exposure to Phenanthrene Promotes Antimicrobial Resistance Genes and Hemolysin Virulence Factor in Guts of Chicken Embryos
Source: Toxics. 2026 Sep 24;14(10):849. doi: 10.3390/toxics14100849 (PMC13709334; doi:10.3390/toxics14100849)
Supplement: Supplementary file 1 [file toxics-14-00849-s001.zip › toxics-4534939-supplementary.pdf]

Supplementary materials

In ovo exposure to phenanthrene promotes antimicrobial resistance genes and hemolysin virulence factor in guts of chicken embryos

Yaoxin Wang [1,2], Wenqian Yu [1,2], Kazuki Kanda [3], Hisato Iwata [3] and Jiahua Guo [1,2*]

[1] Xi'an Key Laboratory of Environmental Simulation and Ecological Health in the Yellow River Basin, College of Urban and Environmental Sciences, Northwest University, Xi'an, 710127, China.

[2] Shaanxi Key Laboratory of Earth Surface System and Environmental Carrying Capacity, College of Urban and Environmental Sciences, Northwest University, Xi'an, 710127, China

[3] Center for Marine Environmental Studies, Ehime University, Matsuyama, 790-8577, Japan

* Correspondence: +86 18992338259. Email: jiahua_guo@nwu.edu.cn

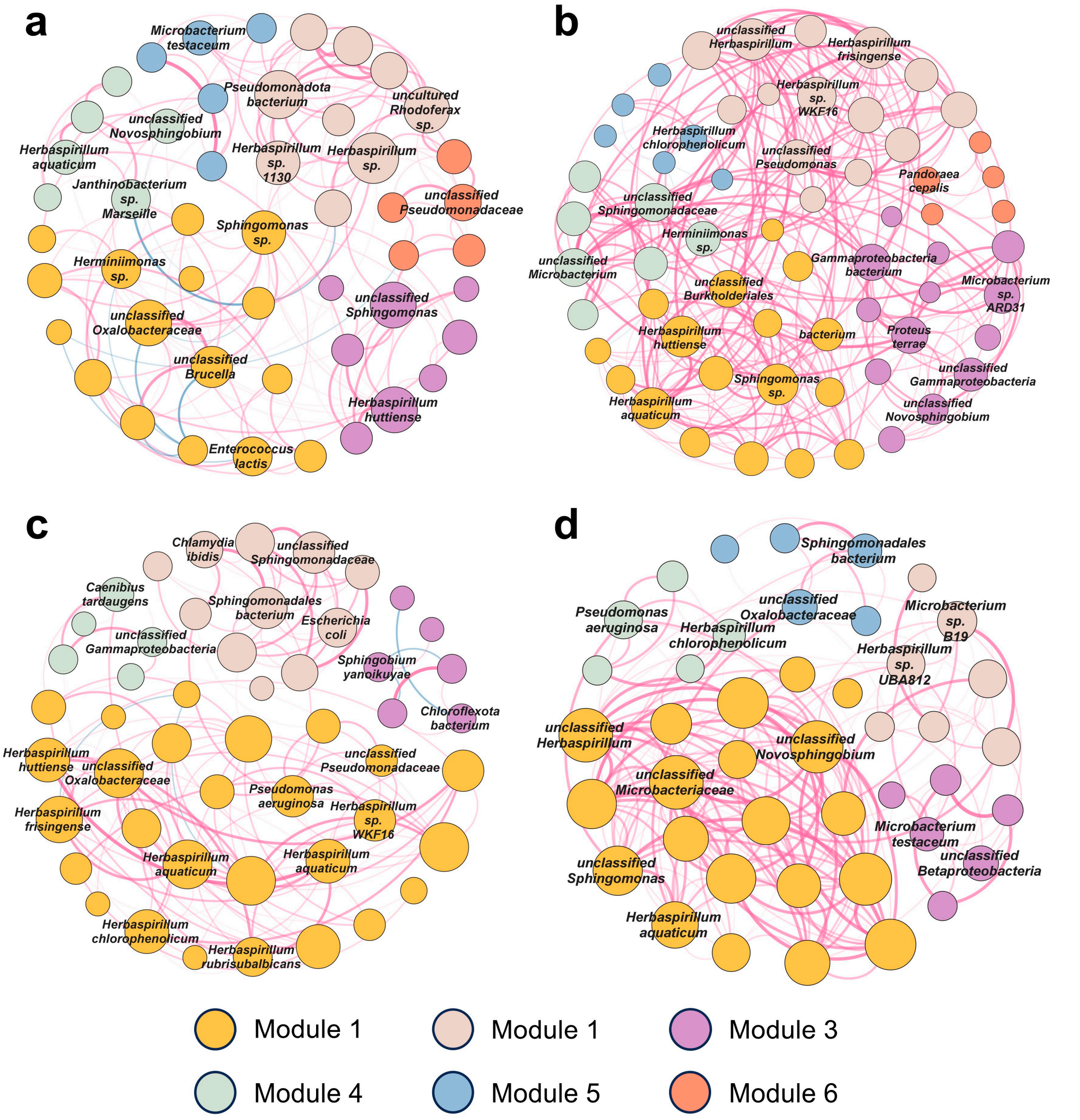

Figure S1. Co-occurrence patterns of the intestinal microbiota of chicken embryos in (a) Control,

(b) Low, (c) Medium and (d) High groups. The width of connecting lines indicates the magnitude of correlation, and the size of each node is proportional to the degree of connectivity. Only significant and robust Spearman correlations are displayed in the network (|R| > 0.8, p < 0.05).

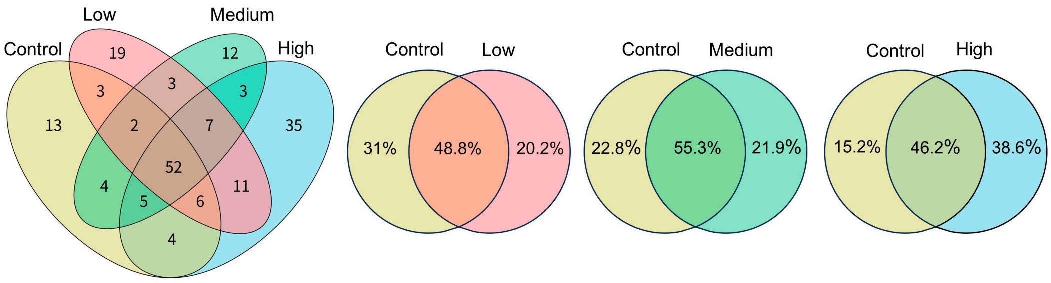

Figure S2. Venn diagram comparison of the number of ARGs.

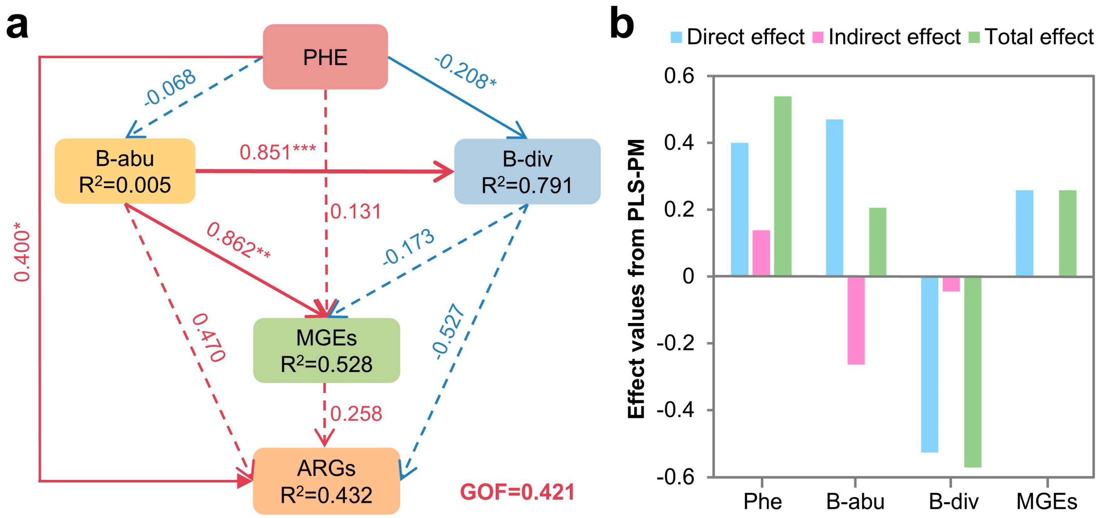

Figure S3. PLS-PM analysis of ARG abundance drivers. (a) Pathways linking PHE, bacterial community, bacterial diversity, MGEs, and ARGs. Positive and negative effects are indicated by red and blue, respectively. R^2 values represent the explained variance. Abbreviations: B-abu, bacterial abundance; B-div, bacterial diversity. (*p < 0.05, **p < 0.01, ***p < 0.001). (b) Effect sizes of the PLS-PM model.
